# SARS-CoV-2 Host Receptor ACE2 Protein Expression Atlas in Human Gastrointestinal Tract

**DOI:** 10.3389/fcell.2021.659809

**Published:** 2021-06-11

**Authors:** Xiang An, Wenlong Lin, Huan Liu, Weixiang Zhong, Xiuming Zhang, Yimin Zhu, Xiaojian Wang, Jun Li, Qinsong Sheng

**Affiliations:** ^1^Institute of Immunology and Bone Marrow Transplantation Center, The First Affiliated Hospital, School of Medicine, Zhejiang University, Hangzhou, China; ^2^Department of Pathology, The First Affiliated Hospital, School of Medicine, Zhejiang University, Hangzhou, China; ^3^Department of Epidemiology and Biostatistics, School of Public Health, Zhejiang University, Hangzhou, China; ^4^Department of Colorectal Surgery, The First Affiliated Hospital, School of Medicine, Zhejiang University, Hangzhou, China

**Keywords:** ACE2, gastrointestinal, immunohistochemistry, gastrointestinal cancers, prognosis

## Abstract

**Background:**

Severe acute respiratory syndrome coronavirus 2 (SARS-CoV-2) infects host cells through interactions with its receptor, Angiotensin-converting enzyme 2 (ACE2), causing severe acute respiratory syndrome and death in a considerable proportion of people. Patients infected with SARS-CoV-2 experience digestive symptoms. However, the precise protein expression atlas of ACE2 in the gastrointestinal tract remains unclear. In this study, we aimed to explore the ACE2 protein expression pattern and the underlying function of ACE2 in the gastrointestinal tract, including the colon, stomach, liver, and pancreas.

**Methods:**

We measured the protein expression of ACE2 in the gastrointestinal tract using immunohistochemical (IHC) staining with an ACE2-specific antibody of paraffin-embedded colon, stomach, liver, and pancreatic tissues. The correlation between the protein expression of ACE2 and the prognosis of patients with gastrointestinal cancers was analyzed by the log-rank (Mantel–Cox) test. The influence of ACE2 on colon, stomach, liver, and pancreatic tumor cell line proliferation was tested using a Cell Counting Kit 8 (CCK-8) assay.

**Results:**

ACE2 presented heterogeneous expression patterns in the gastrointestinal tract, and it showed a punctate distribution in hepatic cells. Compared to that in parallel adjacent non-tumor tissues, the protein expression of ACE2 was significantly increased in colon cancer, stomach cancer, and pancreatic cancer tissues but dramatically decreased in liver cancer tissues. However, the expression level of the ACE2 protein was not correlated with the survival of patients with gastrointestinal cancers. Consistently, ACE2 did not affect the proliferation of gastrointestinal cancer cells *in vitro*.

**Conclusion:**

The ACE2 protein is widely expressed in the gastrointestinal tract, and its expression is significantly altered in gastrointestinal tumor tissues. ACE2 is not an independent prognostic marker of gastrointestinal cancers.

## Introduction

The recent outbreak of novel coronavirus disease 2019 (COVID-19), which is caused by severe acute respiratory syndrome coronavirus 2 (SARS-CoV-2), has become a pandemic and a threat to global health (Wang C. et al., 2020; Zhu N. et al., 2020). As of April 18, 2021, over 140 million people have been infected, and over 3.0 million people have died from COVID-19 due to its severe symptoms^1^, including severe pneumonia, acute respiratory distress, and organ failure driven by hyperinflammation and cytokine storm syndrome (Huang et al., 2020; Wang D. et al., 2020; Wolfel et al., 2020), with a much larger number of infected people suffering worldwide. To date, there is still a lack of efficient drugs for treatment, and little is known about the details of SARS-CoV-2 virus infection, spread, and its immunogenicity.

Angiotensin-converting enzyme 2 (ACE2) was identified as the key human host receptor for SARS-CoV-2 infection. ACE2 has an extracellular-facing N-terminal domain and a C-terminal transmembrane domain with a cytosolic tail. The N-terminal portion of ACE2 contains the claw-like protease domain (PD), while the C-terminal domain is referred to as the collectrin-like domain. The receptor-binding domain (RBD) of SARS-CoV-2 binds to the PD of ACE2, forming the RBD–PD complex, which is distinct from the ACE2 catalytic site (Lan et al., 2020; Wang Q. et al., 2020). The RBD–PD complex is endocytosed into the cytoplasm, facilitating the entry of SARS-CoV-2 into host cells and ultimately resulting in the development of multiorgan damage from SARS-CoV-2 infection (Gheblawi et al., 2020).

Recent clinical case reports showed that some patients infected with SARS-CoV-2 experienced digestive symptoms (Ding and Liang, 2020; Tan et al., 2020; Xu Y. et al., 2020), such as abdominal pain, diarrhea, appetite loss, nausea, vomiting, and blood glucose disorders (Zhu L. et al., 2020), indicating a possibility that the SARS-CoV-2 virus was transmitted among patients with COVID-19 through the fecal–oral route (Ding and Liang, 2020; Jin et al., 2020). Furthermore, it has been reported that the test results for viral SARS-CoV-2 RNA remain positive in feces even after the test results in the respiratory tract become negative. This indicates that viral gastrointestinal infection and potential fecal–oral transmission can last even after viral clearance from the respiratory tract (Xiao et al., 2020), which makes gastroenterology clinical management more difficult during cases of SARS-CoV-2 infection (Sultan et al., 2020).

Several previous studies reported ACE2 expression in the human gastrointestinal tract by IHC staining of limited samples or single-cell RNA sequencing (Hamming et al., 2004; Qi et al., 2020). However, the expression pattern and intracellular location of ACE2 in the gastrointestinal tract is still not clear. Here, we performed a systematic study on the expression of ACE2 in the gastrointestinal tract, utilizing a large sample size. Furthermore, we demonstrated altered expression of ACE2 in digestive tumors and clarified its role in the proliferation of gastrointestinal tract tumor cells.

## Materials and Methods

### Ethics

The experimental use of human paraffin-embedded colon, gastric, pancreatic, and liver cancer sections was approved by the Medical Research Ethics Committee of Zhejiang University (ethics number: ZJU2015-040-01). In addition, informed consent was obtained from all of the subjects involved, and the experiments were conducted according to the principles expressed in the Declaration of Helsinki.

### Immunohistochemical Staining

We obtained a tissue microarray (TMA) of tumor tissues and adjacent non-tumor tissues from the First Affiliated Hospital and Sir Run Run Shaw Hospital of Zhejiang University, School of Medicine, and the National Human Genetic Resources Sharing Service Platform (2005DKA21300). This study included four types of gastrointestinal cancers: colon cancer (409 patients), gastric cancer (254 patients), pancreatic cancer (341 patients), and liver cancer (397 patients). The clinical characteristics of the patients are shown in Supplementary Tables 1–4. Tumor–node–metastasis (TNM) staging according to the American Joint Committee on Cancer (AJCC) 8th Edition Cancer Staging is shown in Supplementary Table 5. All of the corresponding adjacent non-tumor tissues were located more than 2.5 cm away from the tumor tissues and were diagnosed as non-tumor tissues under a microscope by professional pathologists. The IHC assay was performed in the Clinical Laboratory of the First Affiliated Hospital of Zhejiang University, School of Medicine.

The IHC samples were evaluated independently by three researchers (including one pathologist), and the IHC intensity of ACE2 was obtained based on the average score from three researchers. The IHC staining scores of ACE2 were based on the positive area (0–3 score) and intensity (0–4 score) of IHC staining. The ACE2 intensity was then divided into four levels by multiplying the positive area score and the intensity score: negative (score = 0), weak (0 < score ≤ 4), moderate (4 < score ≤ 8), and strong (8 < score ≤ 12).

### Cell Culture and Reagents

SW480, Huh7, HGC, and CFPAC1 cells were obtained from the ATCC. The cells were cultured at 37°C under 5% CO_2_ in Dulbecco’s modified Eagle’s medium (DMEM) or RPMI 1640 containing 10% FBS, 100 U ml^–1^ penicillin, and 100 μg ml^–1^ streptomycin. The anti-ACE2 antibodies used in the IHC staining and western blotting were obtained from Abcam (ab108252). Ang (1–7) was obtained from MedChemExpress (HY-12403).

### Generation and Validation of ACE2 KO Cells

Angiotensin-converting enzyme 2-knockout (KO) cells were constructed using the CRISPR/Cas9 gene-editing system. The CRISPR plasmid pEP-330x (Addgene) contains expression cassettes of Cas9 and puromycin resistance genes. The target sequences of the gRNAs were designed using the MIT online tool^2^. To generate the ACE2-KO cells, two gRNAs (gRNA #1, forward: ACCGTTACATATCTGTCCTCTCC, reverse: AACGGAGAGGACAGATATGTAAC; gRNA #2, forward: ACCGTGAGTTCTCATGGCTCTAT, reverse: AACATAGAGCCATGAGAACTCAC) targeting the exons of ACE2 were designed and inserted into the pEP-330x vector by using the *Bpi*I (Thermo) site and then cotransfected into SW480/Huh7 cells using Lipofectamine 3000 (Invitrogen, #L3000001). Forty-eight hours after transfection, the cells were treated for 72 h with puromycin (Sigma-Aldrich) and allowed to recover and then seeded as single cells in 96-well plates. The KO efficiency was detected by western blotting.

### Western Blot Analysis

The cells were lysed with cell lysis buffer (Cell Signaling Technology) supplemented with a protease inhibitor cocktail (Sigma-Aldrich). Protein concentrations in the extracts were measured with a BCA protein assay kit (Pierce) and were adjusted to be equal. The cell lysates were loaded and subjected to SDS-PAGE, transferred onto nitrocellulose membranes, and then analyzed with the indicated antibodies: anti-ACE2 (Abcam, ab108252, 1:1,000 dilution) and anti-β-actin (Proteintech, 66009-1-Ig, 1:2,000 dilution).

### RNA Isolation and Real-Time Quantitative PCR

Total RNA was extracted using the TRIzol reagent (Life Technologies) according to the manufacturer’s instructions. Reverse transcription was performed using Reverse Transcriptase M-MLV (TaKaRa) according to the manufacturer’s instructions. Real-time quantitative PCR (qRT-PCR) analysis was performed using SYBR Green Master Mix (Yeasen) on a CFX Touch system (Bio-Rad), and GAPDH was used as an internal control. The PCR products were subjected to agarose gel electrophoresis. The primer sequences were as follows: *MAS1*, forward TCACCCACCTGTCTATCGCA, reverse CACTAATGGCCGT CAGCAGAT; *GAPDH*, forward GAGTCAACGGATTTGGT CGT, reverse GACAAGCTTCCCGTTCTCAG.

### Cell Proliferation Assay

A total of 3 × 10^5^ cells were seeded into 12-well plates and then transfected with siRNA oligos, including a negative control (siNC, UUCUCCGAACGUGUCACGUTT) or specific targeting of human ACE2 (siACE2; #1, GCGAGUGGCUAAUUUGAA ATT; #2, GCACUUUGUCAAGCAGCUATT; #3, GGACAA GUUUAACCACGAATT). Twenty-four hours later, the cells were reseeded into 96-well plates at 3 × 10^3^ per well. The cells were serum starved for 12 h, followed by serum stimulation with or without Ang (1–7) (100 nM). Ten microliters of Cell Counting Kit 8 (CCK-8) was added to each well, and they were cultured for another 4 h. Their absorbance at 450 nm was measured, and their proliferation was calculated.

### Statistical Analysis

Statistical analyses were performed with SPSS 20.0 (IBM Corporation) and GraphPad Prism v5.0 (GraphPad software). The relationships between the clinical variables and ACE2 IHC intensity were analyzed by the Kruskal–Wallis test (Li et al., 2019). Kaplan–Meier survival analysis was performed using the log-rank (Mantel–Cox) test. To compare the ACE2 IHC intensity between lower-age and higher-age patients, the patients were divided into two groups—a lower age (<65 years) and a higher age (≥65 years) (Daste et al., 2017)—and the statistical significance between the two groups was calculated using unpaired Student’s *t*-tests. A *p*-value < 0.05 was considered statistically significant (^∗^*p* < 0.05; ^∗∗^*p* < 0.01; ^∗∗∗^*p* < 0.001; NS, no significance, *p* > 0.05).

## Results

### Expression Patterns of ACE2 in the Gastrointestinal Tract

The highest level of expression of ACE2 mRNA was observed in the intestine (Supplementary Figure 1A). According to the Human Protein Atlas database^3^ (Uhlén et al., 2015), ACE2 was expressed in the intestine and colon but not in the pancreas, liver, or stomach (Supplementary Figure 1B). However, the sample size in the Human Protein Atlas was less than 10 and thus not sufficient to construct a detailed expression map of ACE2 in the gastrointestinal tract. To fully understand the precise ACE2 location and expression patterns in the gastrointestinal tract, we collected gastrointestinal tissues from patients with cancers, which were diagnosed as adjacent non-tumor tissues by a professional pathologist *via* hematoxylin and eosin (H&E) staining. IHC staining of ACE2 was performed in TMAs, including 409 cases of colon cancer, 232 cases of stomach cancer, 131 cases of pancreatic cancer, and 72 cases of liver cancer and adjacent non-tumor tissues.

**FIGURE 1:**
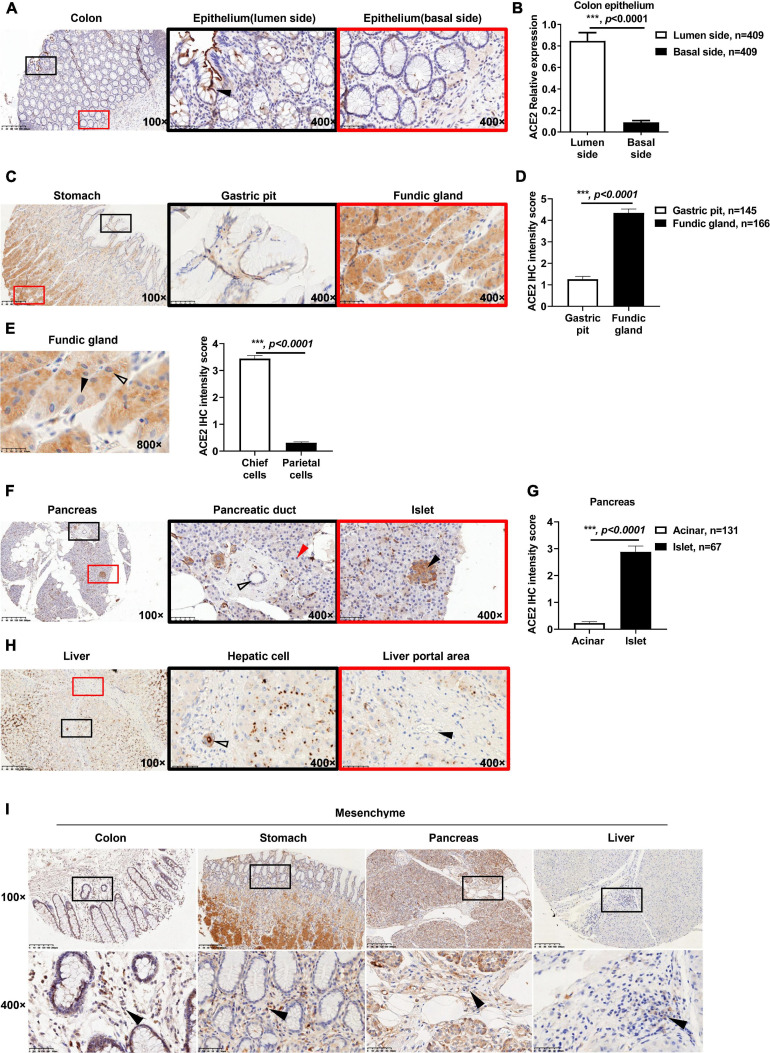
Expression patterns of ACE2 in gastrointestinal tissues. **(A)** Representative immunohistochemical (IHC) staining of human colon sections with anti-ACE2 antibody. The black and red boxes are the indicated 400× fields in the 100× sections (black arrow: goblet cell). **(B)** Comparison of the ACE2 IHC intensity score between the basal side and lumen side of the colon epithelium. **(C)** Representative IHC staining of human stomach sections with the anti-ACE2 antibody. **(D)** Comparison of ACE2 expression between the gastric pit and fundic gland. **(E)** ACE2 expression in chief cells was higher (hollow arrow) than that in parietal cells (black arrow). Four chief cells and four parietal cells were analyzed per sample (50 samples). **(F)** Representative IHC staining of ACE2 protein in the pancreas, acinar cells (red arrow), pancreatic duct (hollow arrow), and pancreatic islet (black arrow). **(G)** Comparison of ACE2 IHC intensity between pancreatic acinar cells and pancreatic islets. **(H)** Representative IHC staining of human liver sections with the anti-ACE2 antibody. Interlobular duct (hollow arrow) and interlobular vessel (black arrow). **(I)** Representative IHC staining of gastrointestinal tissues. The black boxes are the indicated 400× fields in the 100× sections, and the black arrow indicates mesenchymal cells. Scale bar, 200 μm (100×) and 50 μm (400×). Data are shown as the mean ± SEM **(B,D)**.

As described in the methods, the ACE2 IHC intensity was divided into four levels by multiplying the positive area score and the intensity score: negative (score = 0), weak (0 < score ≤ 4), moderate (4 < score ≤ 8), and strong (8 < score ≤ 12). As shown in Supplementary Figure 2A, the colon tissues showed negative to moderate staining for ACE2. In brief, 68.9% (282/409) of the colon biopsies were negative for ACE2 staining, 27.6% (113/409) of the biopsies showed weak ACE2 staining, and 3.5% (14/409) of the biopsies showed moderate ACE2 staining. Of note, positive ACE2 staining was mainly located on the membrane and cytoplasm of the goblet cells (Figure 1A and Supplementary Figure 2B). Moreover, ACE2 staining on the lumen side of the colon epithelium was stronger than that on the basal side (Figure 1B).

It has been reported that some patients infected with SARS-CoV-2 experience digestive symptoms (Ding and Liang, 2020; Tan et al., 2020; Xu Y. et al., 2020), including appetite loss. However, little is known about the details of ACE2 expression in the stomach. From the IHC staining results of ACE2 in the stomach, a complicated expression atlas of ACE2 was set up. Briefly, in 145 biopsies containing gastric pits, 37.9% (55/145) of biopsies were negative for ACE2 staining, 57.9% (84/145) of biopsies showed weak ACE2 staining, and only 4.2% (6/145) of biopsies were moderate for ACE2 staining (Supplementary Figure 2C). The fundic gland showed weak to moderate ACE2 staining intensity. In 166 biopsies containing fundic glands, 62.7% (104/166) of the biopsies showed weak ACE2 staining, and 37.3% (62/166) of biopsies were moderate for ACE2 staining (Supplementary Figure 2D). The fundic gland showed a higher expression level of ACE2 staining than the gastric pit (Figures 1C,D and Supplementary Figure 2E). Furthermore, gastric chief cells (pepsinogen-secreting cells) expressed higher ACE2 levels in the cytoplasm than did parietal cells (gastric acid-secreting cells) (Figure 1E). Since most biopsies were obtained from patients with gastric cancer, only one biopsy contained pyloric glands, which showed weak ACE2 staining (Supplementary Figure 2F).

In the pancreas, 95% of pancreatic tissue is exocrine tissue that produces pancreatic enzymes for digestion, which are also called acinar cells. The remaining tissue consists of endocrine cells called islets of Langerhans, also named islets. Based on the ACE2 IHC staining intensity, we first demonstrated that acinar cells showed negative or weak ACE2 staining, while the lumen side of the pancreatic duct cells showed negative to moderate ACE2 staining (Figure 1F and Supplementary Figure 2G). Briefly, 84.7% (111/131) of biopsies were negative for ACE2 staining in the acinar cells, and 15.3% (20/131) of biopsies showed weak ACE2 staining (Supplementary Figure 2H). In 104 biopsies containing pancreatic ducts, 34.6% (36/104) of biopsies were negative for ACE2 staining, 62.5% (65/104) of biopsies showed weak ACE2 staining, and only 2.9% (3/104) of biopsies showed moderate ACE2 staining (Supplementary Figure 2I). Moreover, in 67 pancreatic tissues that contained islets, 83.6% (56/67) of biopsies showed weak ACE2 staining, while 16.4% (11/67) of biopsies showed moderate ACE2 staining in the cytoplasm of islet cells (Supplementary Figure 2J). ACE2 staining was stronger in islet cells than in acinar cells (Figures 1F,G).

Interestingly, ACE2 was granularly distributed in the cytoplasm of hepatocytes (Figure 1H and Supplementary Figure 2K), which was significantly different from its expression patterns in the colon, stomach, and pancreas. Of all 72 biopsies, 12.5% (9/72) were negative for ACE2 staining, 77.8% (56/72) showed weak ACE2 staining, and 9.7% (7/72) showed moderate ACE2 staining (Supplementary Figure 2L). In the portal area of the liver, the lumen side of the interlobular bile duct cells (cholangiocytes) showed negative to strong ACE2 staining (Figure 1H and Supplementary Figure 2M). In detail, 3.8% (2/53) of biopsies were negative for ACE2 staining, 75.4% (40/53) of biopsies showed weak ACE2 staining, 17% (9/53) of biopsies showed moderate ACE2 staining, and 3.8% (2/53) of biopsies showed strong ACE2 staining, while the interlobular vessels were all negative (100%, 53/53) (Figure 1H).

The protein expression of ACE2 was also observed in the cytoplasm of mesenchymal cells in all of colon (409/409), stomach (145/145), and pancreatic (104/104) tissues (Figure 1I). In 53 liver biopsies containing connective tissue, only 32.1% (17/53) of biopsies showed positive ACE2 staining in mesenchymal cells; however, the exact cell type is still unknown. Intriguingly, the capillary endothelial cells among the acinar cells were almost all positive (98.5%, 129/131) for ACE2 staining. In detail, 1.5% (2/131) of biopsies were negative for ACE2 staining, 92.4% (121/131) of biopsies showed weak ACE2 staining, 4.6% (6/131) of biopsies showed moderate ACE2 staining, and 1.5% (2/131) of biopsies showed strong ACE2 staining (Supplementary Figures 2N,O). However, all capillary endothelial cells in the colon (409/409), stomach (129/129), and liver (53/53) were negative for ACE2 staining (Supplementary Figure 2P).

Overall, ACE2 presented heterogeneous expression patterns in the gastrointestinal tract, which may impact the susceptibility of these organs to SARS-CoV-2 infection.

### Altered Protein Expression of ACE2 in Gastrointestinal Tumors

**FIGURE 2:**
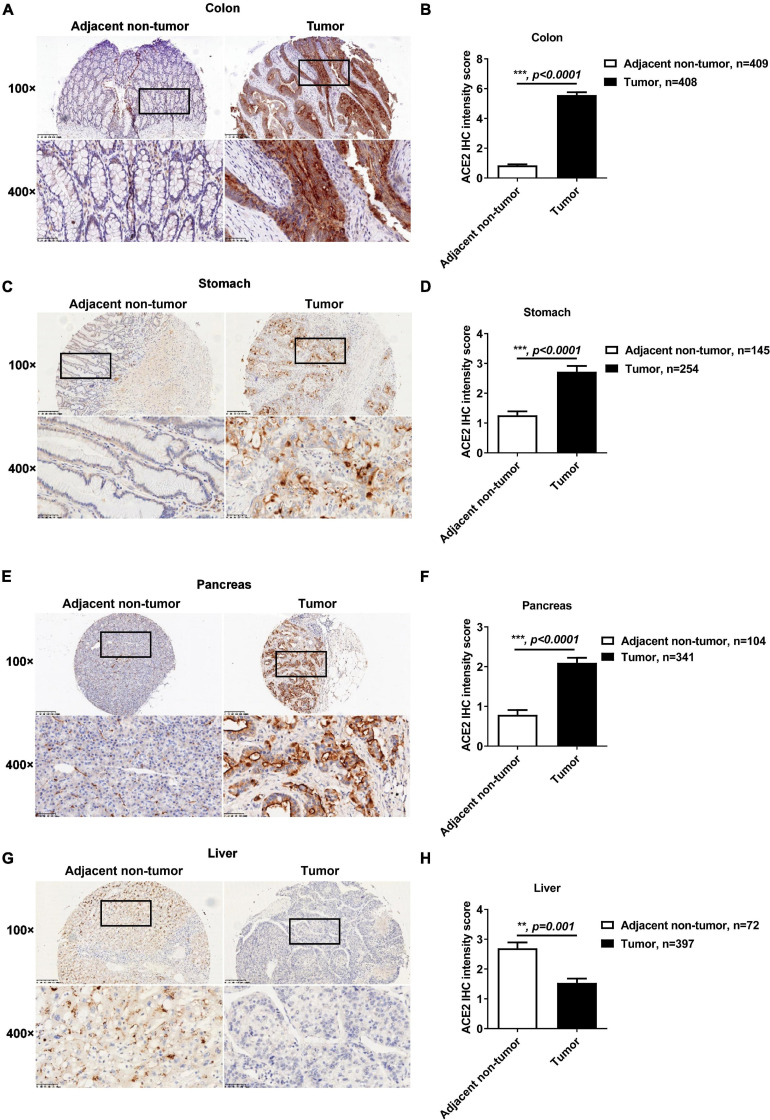
The protein expression of ACE2 was increased in colon, gastric, and pancreatic cancer but decreased in liver cancer. The protein expression of ACE2 in colon cancer, gastric cancer, pancreatic cancer, and liver cancer tissues and their adjacent non-tumor tissues was determined by IHC. **(A,B)** Increased ACE2 expression in colon cancer tissues. **(C,D)** Increased ACE2 expression in gastric cancer tissues. **(E,F)** Increased ACE2 expression in pancreatic cancer tissues. **(G,H)** Decreased ACE2 expression in liver cancer tissues. Scale bar, 200 μm (100×) and 50 μm (400×). Data are shown as the mean ± SEM **(B,D,F,H)**.

Previous reports have demonstrated that cancers are SARS-CoV-2 infection risk factors (Dai et al., 2020; Wang and Zhang, 2020). We therefore explored the expression profiles of ACE2 in gastrointestinal cancers. As shown in Supplementary Figure 3A, the mRNA expression of ACE2 was significantly increased in colon adenocarcinoma, stomach adenocarcinoma, and pancreatic adenocarcinoma compared to their corresponding adjacent non-tumor tissues. The mRNA expression of ACE2 was comparable in liver tumor tissues and normal liver tissues. Consistently, the median staining intensity score of ACE2 in colon epithelial cells was significantly increased in tumor tissues (5.57 ± 0.19) compared with adjacent non-tumor tissues (0.85 ± 0.08) (Figures 2A,B). The protein intensity score of ACE2 staining in gastric tumor tissues (2.72 ± 0.19) was also significantly upregulated compared to that in adjacent non-tumor gastric tissues (1.26 ± 0.14) (Figures 2C,D). Compared to duct epithelial cells from adjacent non-tumor pancreas tissues (0.93 ± 0.12), pancreatic cancer tissues showed an enhanced intensity score of ACE2 staining (2.14 ± 0.12) (Figures 2E,F). However, the protein intensity score of ACE2 staining was decreased in liver tumor tissues (1.54 ± 0.14) compared to adjacent non-tumor liver tissues (2.69 ± 0.20) (Figures 2G,H).

Considering that ACE2 is located on the *X*-chromosome, we compared the protein expression level of ACE2 between males and females. As shown in Supplementary Figures 3B,C, there was no significant difference in ACE2 protein expression between males and females in the four gastrointestinal tumor tissues (Supplementary Figure 3B) or in their corresponding adjacent non-tumor tissues (Supplementary Figure 3C).

In addition to sex, the ACE2 expression level in gastrointestinal tissues was compared between lower-age patients (<65 years) and higher-age patients (≥65 years) (Daste et al., 2017). ACE2 expression was significantly higher in colon cancer tissues from higher-age patients than in tissues from lower-age patients (Supplementary Figure 4A). However, when comparing lower-age gastric, pancreatic, and liver cancer patients, the expression of ACE2 was similar in the cancer tissues and their corresponding adjacent non-tumor tissues (Supplementary Figures 4A,B). No age association with the ACE2 protein levels was found (Supplementary Figures 4C,D).

Consistently, based on the clinical characteristics of patients with gastrointestinal cancers (Supplementary Tables 1–4), we found significant differences in ACE2 expression levels between subgroups of colon cancer patients with different metastasis (M) stages (*p* = 0.03) and TNM stages (*p* = 0.021) (Supplementary Table 1). ACE2 expression also showed significant differences between subgroups of gastric cancer patients with different tumor invasion depth (T) stages (*p* = 0.004) (Supplementary Table 2). However, the expression level of ACE2 was not associated with the clinical characteristics of pancreatic cancer patients (Supplementary Table 3). In addition, the ACE2 expression level showed significant differences between subgroups of liver cancer patients with different lymph node metastasis stages (*p* = 0.02) (Supplementary Table 4).

Taken together, these results indicated that both the protein and mRNA expressions of ACE2 were significantly altered in gastrointestinal tumors relative to normal tissues.

### Protein Expression Patterns of ACE2 in Tumor Mesenchymal Tissue and Intestinal Metaplasia

**FIGURE 3:**
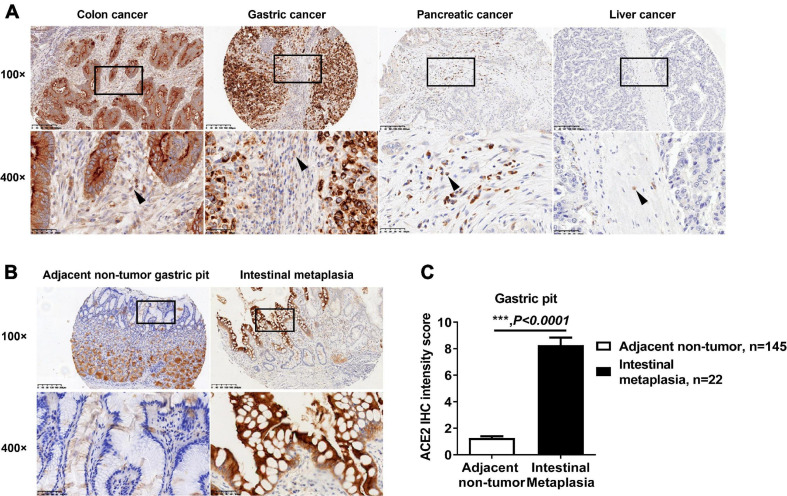
Expression pattern of ACE2 in intestinal metaplasia and tumor mesenchymal tissue. **(A)** Representative IHC staining of human gastrointestinal tumor sections with anti-ACE2 antibody. The black boxes are the indicated 400× fields in the 100× sections, and the black arrow indicates mesenchymal cells. **(B)** Representative IHC staining of human gastric pit sections with anti-ACE2 antibody. The black boxes are the indicated 400× fields in the 100× sections. **(C)** Comparison of the ACE2 IHC intensity score between intestinal metaplasia and gastric pit cells. Scale bar, 200 μm (100×) and 50 μm (400×). Data are shown as the mean ± SEM.

Mesenchymal tissue cells in the gastrointestinal tract play a key role in carcinogenesis (Koliaraki et al., 2017). However, the expression of ACE2 in these cells remains unclear. All colon cancer biopsies (408/408), gastric cancer biopsies (254/254), and pancreatic cancer biopsies (341/341) showed ACE2-positive staining in mesenchymal cells (Figure 3A). However, in 134 liver cancer biopsies containing connective tissue, only 28 biopsies were positive for ACE2 staining (Figure 3A). Gastric intestinal metaplasia (IM) is considered to be a critical premalignant lesion of gastric cancer (Jencks et al., 2018). In our TMAs, 22 adjacent non-tumor gastric biopsies were diagnosed as IM and showed enhanced intensity of ACE2 staining when compared with that in other adjacent non-tumor gastric pits (Figures 3B,C), which is consistent with a previous report that the expression of ACE2 is significantly upregulated in IM (Lee et al., 2010).

### Protein Expression Levels of ACE2 Are Not Correlated With the Survival of Patients With Gastrointestinal Cancers

**FIGURE 4:**
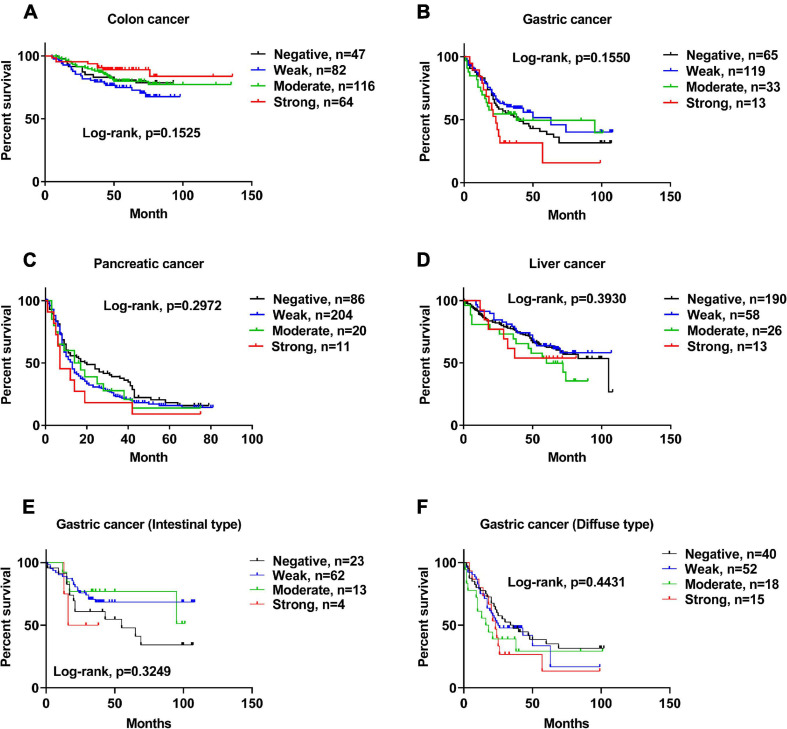
The protein expression level of ACE2 was not correlated with the survival of patients with gastrointestinal cancers. **(A–F)** Kaplan–Meier analysis of overall survival in the set of patients with colon cancer **(A)**, gastric cancer **(B)**, pancreatic cancer **(C)**, liver cancer **(D)**, intestinal-type gastric cancer **(E)**, and diffuse-type gastric cancer **(F)**.

Based on the detailed survival information and ACE2 IHC staining of patients, we demonstrated that the protein expression levels of ACE2 were not correlated with the survival of patients with colon cancer (Figure 4A), gastric cancer (Figure 4B), pancreatic cancer (Figure 4C), or liver cancer (Figure 4D). Consistently, ACE2 expression was not associated with the Lauren classification of gastric cancer (Figures 4E,F and Supplementary Table 2).

### ACE2 Does Not Affect the Proliferation of Gastrointestinal Tumor Cell Lines

**FIGURE 5:**
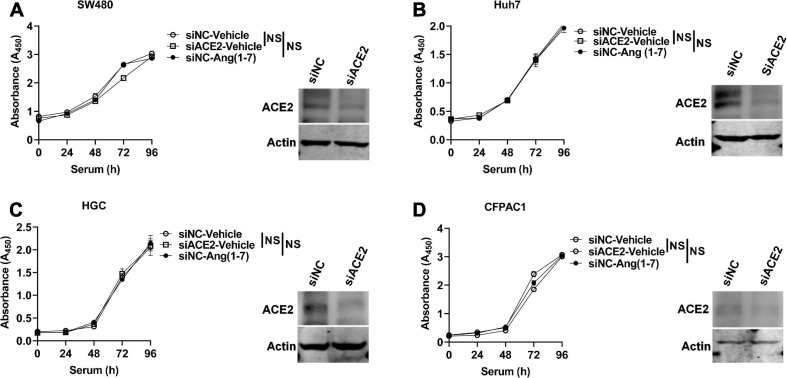
ACE2 does not affect the proliferation of gastrointestinal tumor cell lines. ACE2 was knocked down using siRNA oligos specifically targeted against human ACE2 (siACE2) or negative control (siNC) in gastrointestinal tumor cell lines, and cell proliferation was detected by using the CCK-8 method. **(A–D)** Proliferation of ACE2 knockdown (siACE2) and negative control (siNC) SW480 **(A)**, Huh7 **(B)**, HGC **(C)**, and CFPAC1 **(D)** cells stimulated with serum with or without Ang (1–7) for the indicated times. Western blotting confirmed the ACE2 knockdown efficiency in the cells. Data are shown as the mean ± SEM and are representative of three independent replicates. NS, no significance.

Ang (1–7), a heptapeptide converted from Ang I by ACE and from Ang II by ACE2, binds to Mas (MAS, coded by *MAS1*) and inhibits proliferation of prostate cancer lines (Krishnan et al., 2013). However, Ang (1–7) has no influence on the cell cycle in human colon adenocarcinoma cells (Bernardi et al., 2012). It has been reported that ACE2 expression is associated with tumor progression and that silencing ACE2 expression promotes cell proliferation in breast cancer (Yu et al., 2016; Zhang et al., 2019). Therefore, we explored whether ACE2 could affect the proliferation of gastrointestinal tumor cell lines. Before the measurement of cell proliferation, the gene expression of *MAS1* was determined. As shown in Supplementary Figure 5A, *MAS1* was expressed in all of these cells. ACE2 was knocked down in gastrointestinal tumor cell lines, including the colon cancer cell line SW480, liver cancer cell line Huh7, gastric cancer cell line HGC, and pancreatic cancer cell line CFPAC1, using RNA interference oligos (RNAi) targeting human ACE2, and the proliferation effects upon serum supplementation were detected by a CCK-8 assay. As shown in Figure 5, knockdown of ACE2 had no effect on the proliferation of SW480 (Figure 5A), HepG2 (Figure 5B), HGC (Figure 5C), or CFPAC1 cells (Figure 5D). Likewise, ACE2 knockout did not affect SW480 (Supplementary Figure 5B) or Huh7 cell proliferation (Supplementary Figure 5C). Consistently, Ang (1–7) treatment did not affect the proliferation of any of the above cell lines (Figures 5A–D).

## Discussion

Coronavirus disease 2019 is a global health crisis, and many efforts are still needed to control SARS-CoV-2 spread among humans and to reduce the mortality of infected patients, especially among patients who have conditions such as tumors, cardiovascular diseases, and metabolic diseases.

Here, we systemically showed that the key host receptor protein ACE2 is widely expressed in the digestive system with different expression patterns, which may explain why some patients infected with SARS-CoV-2 experienced gastrointestinal symptoms during COVID-19 pathogenesis.

Furthermore, the protein expression of ACE2 in gastrointestinal cancer tissues was significantly altered compared to that in adjacent non-tumor tissues. However, no significant correlation was observed between ACE2 protein expression and the prognosis of patients with gastrointestinal cancers.

In a recent COVID-19 cohort, patients with diarrhea had a higher prevalence of detectable stool viral RNA on presentation (Chen et al., 2020; Jin et al., 2020; Tan et al., 2020; Xiao et al., 2020). It has been reported that the ACE2 protein is highly expressed in SARS-CoV-2-infected human gastric, duodenal, and rectum glandular epithelial cells but not in esophageal epithelium (Xiao et al., 2020). Furthermore, SARS-CoV-2 RNA and intracellular staining of viral nucleocapsid protein were detected in the gastric, duodenal, and rectal epithelia of SARS-CoV-2-infected patients. Thus, SARS-CoV-2 was thought to mainly infect these gastrointestinal glandular epithelial cells (Xiao et al., 2020). After infection, the SARS-CoV-2 virus undergoes its life cycle mainly in glandular epithelial cells of the colon and stomach, causing irreversible damage to these cells and resulting in gastrointestinal symptoms, such as abdominal pain, diarrhea, appetite loss, nausea, and vomiting. In some patients with COVID-19, gastrointestinal manifestations were the only initial symptoms.

Several previous studies have explored the expression pattern of ACE2 in the human gastrointestinal tract by IHC, RNA sequencing, and single-cell RNA sequencing (Hamming et al., 2004; Li et al., 2020; Qi et al., 2020; Xu J. et al., 2020). Hamming et al. (2004) reported that ACE2 was abundantly expressed in the human small intestine but was negative in human hepatic cells (*n* = 6). After analyzing the single-cell RNA sequencing data of five liver samples from GEO, Qi et al. (2020) reported that ACE2 was mainly expressed in cholangiocytes but had only limited expression in hepatic cells. Based on the Genotype-Tissue Expression (GTEx) project and The Cancer Genome Atlas (TCGA) program, Li et al. (2020) reported abundant ACE2 distribution in the gastrointestinal tract, especially high ACE2 expression in the small intestine and colon. However, these data were based on limited sample sizes (*n* < 10) or contained only mRNA data.

Herein, using a larger clinical sample, we systemically explored the ACE2 protein expression atlas in the human gastrointestinal tract and showed more details. We first demonstrated that ACE2 expression was lower on the basal side than on the lumen side of the colon epithelium (*n* = 409). Given that colon basal side cells are capable of regeneration and differentiation into mature functional glandular epithelial cells, the damaged colon epithelium would be repaired in patients infected with SARS-CoV-2 after the clearance of the SARS-CoV-2 virus, which may explain why the diarrhea of patients with COVID-19 was self-limiting (Jin et al., 2020). In the stomach tissue, we revealed that gastric chief cells (pepsinogen-secreting cells) expressed higher levels of ACE2 in the cytoplasm than parietal cells (gastric acid-secreting cells). Gastric chief cells produce the proenzyme pepsinogen, which is subsequently converted to the acid protease pepsin and plays a key role in gastric digestive function and appetite. This may explain why patients infected with SARS-CoV-2 exhibited a clinical phenotype of appetite loss.

Meanwhile, the ACE2 protein was highly expressed in IM cells in the stomach, which are precursors to gastric carcinogenesis. Consistently, Xu J. et al. (2020) revealed elevated ACE2 expression in stomach IM, and the expression level of ACE2 was increased with the severity of IM.

Single-cell sequencing data have demonstrated that ACE2 mRNA expression was observed in islet and duct system cells but not in the acinar cells of the pancreas (Liu et al., 2020). Here, we demonstrated that 83.6% (56/67) of biopsies were positive for ACE2 staining in islets, while only 15.3% (20/131) of biopsies showed weak ACE2 staining in acinar cells. In addition, 98.5% (129/131) of acinar cells showed positive ACE2 staining in the capillary endothelial cells. SARS-CoV-2 may spread among pancreatic tissues *via* duct system cells and ultimately cause islet damage, resulting in abnormal blood glucose (Aloysius et al., 2020; Liu et al., 2020; Wang F. et al., 2020; Zhu L. et al., 2020). Liu et al. (2020) recently reported that 1 to 2% of non-severe and 17% of severe patients with COVID-19 had pancreatic injury, which is consistent with our notion that specific ACE2 expression in the pancreas results in this phenotype after SARS-CoV-2 infection. Therefore, the highly expressed ACE2 in pancreatic islets may increase the risk of SARS-COV-2 infection and damage (Liu et al., 2020).

Hamming et al. (2004) reported that ACE2 expression was negative in human hepatic cells (*n* = 6). Through analyzing single-cell RNA sequencing data from five liver samples from GEO, Qi et al. (2020) reported that ACE2 was mainly expressed in cholangiocytes but was barely expressed in hepatic cells. Here, we revealed that most liver biopsies (96.2%, 51/53) were positive for ACE2 staining on the lumen side of liver cholangiocytes, which is similar to the report of Qi et al. (2020). However, 87.5% (63/72) of samples were positive for ACE2 staining in hepatic cells. We noticed that ACE2 was granularly distributed in the cytoplasm of hepatic cells, which is consistent with the notion that hepatocytes secrete ACE2 to regulate blood pressure (Herath et al., 2007; Lambert et al., 2008; Ingelfinger, 2009; Tan et al., 2020).

In the intestine, ACE2 regulates the absorption of electrolytes and glucose (Garg et al., 2012). Abnormal ACE2 expression may lead to diarrhea, malnutrition, and other inflammatory diseases. It has been reported that ACE2-deficient mice suffer from more severe symptoms in a dextran sodium sulfate (DSS)-induced experimental colitis model, which occurs not through the renin–angiotensin system (RAS) but through the regulation of intestinal amino acid homeostasis (Hashimoto et al., 2012). Another study came to the opposite conclusion that the ACE2 inhibitor GL1001 attenuated the severity of DSS-induced colitis (Byrnes et al., 2009), and these data indicated that ACE2 may exert a dual role in inflammatory bowel disease (IBD). It has been reported that ACE2-knockout mice exhibit more severe insulin resistance and glucose intolerance in response to a high-fat diet (Xuan et al., 2018). Herath et al. (2007) found that ACE2 suppressed liver injury and fibrosis progression in a murine model (Osterreicher et al., 2009). ACE2 gene expression is altered in many cancers (Chai et al., 2020), and it has been associated with tumor cell proliferation and metastasis, such as pancreatic cancer, breast cancer, lung cancer, and renal cancer (Feng et al., 2010; Zhou et al., 2011; Yu et al., 2016; Narayan et al., 2020; Yang et al., 2020). Here, we demonstrated that ACE2 protein expression was significantly upregulated in colon cancer cells, gastric cancer cells, and pancreatic cancer cells but downregulated in liver cancer cells. However, the altered protein expression of ACE2 was not associated with the prognosis of the patients suffering from gastrointestinal cancers, and it did not affect gastrointestinal cancer cell proliferation.

## Conclusion

In summary, we demonstrated that ACE2 was differentially expressed in complicated cell types of the gastrointestinal tract. Mature gastrointestinal epithelial cells of barrier tissues, such as colonic glandular epithelial cells, are potential targets during SARS-CoV-2 infection due to the high level of ACE2 protein expression in these cells. ACE2 does not affect gastrointestinal cancer cell proliferation. Furthermore, no significant correlation of ACE2 protein expression with the survival of gastrointestinal cancer patients was observed. Taken together, ACE2 acts as a host receptor of SARS-CoV-2 but does not affect the progression of gastrointestinal cancers.

## Data Availability Statement

The original contributions presented in the study are included in the article/Supplementary Material, further inquiries can be directed to the corresponding author/s.

## Ethics Statement

The studies involving human participants were reviewed and approved by the Medical Research Ethics Committee of Zhejiang University. The patients/participants provided their written informed consent to participate in this study.

## Author Contributions

XW designed the experiments. JL, WZ, XZ, and QS provided the samples and expert histopathological analysis. YZ provided guidance for the statistical analyses. XA, WL, and HL conducted cellular studies and analyzed the data. XA, WL, HL, and XW wrote and revised the manuscript, which was edited by all authors. All authors contributed to the article and approved the submitted version.

## Conflict of Interest

The authors declare that the research was conducted in the absence of any commercial or financial relationships that could be construed as a potential conflict of interest.

## Footnotes

1 https://www.who.int/emergencies/diseases/novel-coronavirus-2019

2 http://crispr.mit.edu/

3 https://www.proteinatlas.org/

## Abbreviations

COVID-19: coronavirus disease 2019
SARA-CoV-2: severe acute respiratory syndrome coronavirus 2
ACE2: angiotensin-converting enzyme 2
H&E: hematoxylin and eosin
IHC: immunohistochemical staining
TMAs: tissue microarrays
mRNA: messenger RNA
siRNA: small interference RNA
RNAi: RNA interference oligos
CCK-8: Cell Counting Kit 8.
