# Supplementary material for: SARS-CoV-2 Host Receptor ACE2 Protein Expression Atlas in Human Gastrointestinal Tract
Source: Front Cell Dev Biol. 2021 Jun 11;9:659809. doi: 10.3389/fcell.2021.659809 (PMC8226145; doi:10.3389/fcell.2021.659809)
Supplement: Supplementary file 1 [file Data_Sheet_1.PDF]

A

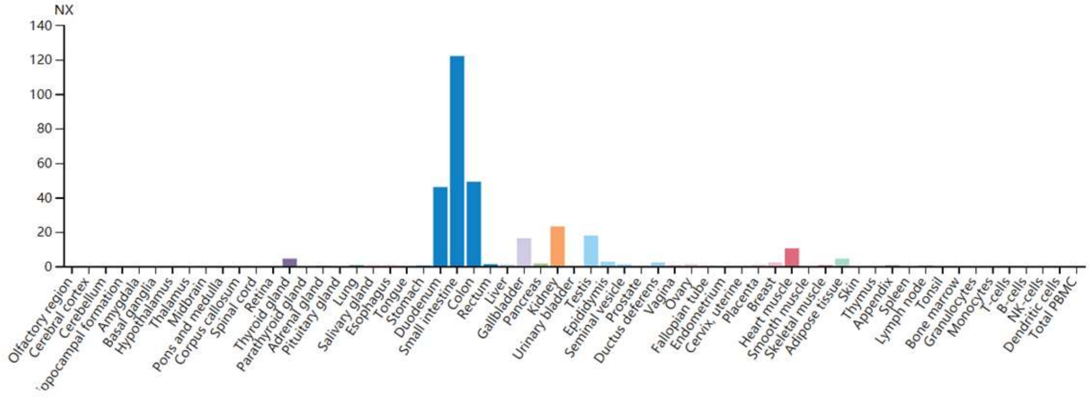

mRNA expression of ACE2

B

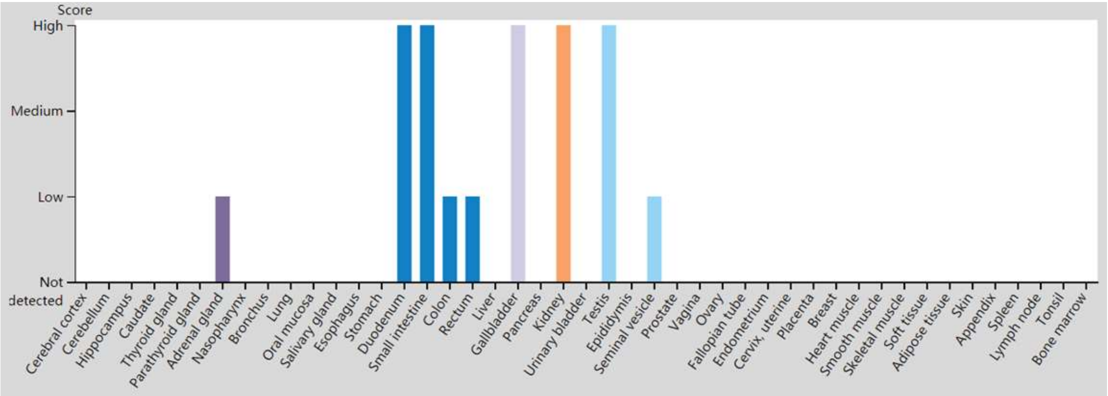

Protein expression of ACE2

Figure S1. mRNA and protein expression atlas of ACE2 in human tissues from HPA database. (A) mRNA expression atlas of ACE2. **(B)** Protein expression atlas of ACE2.

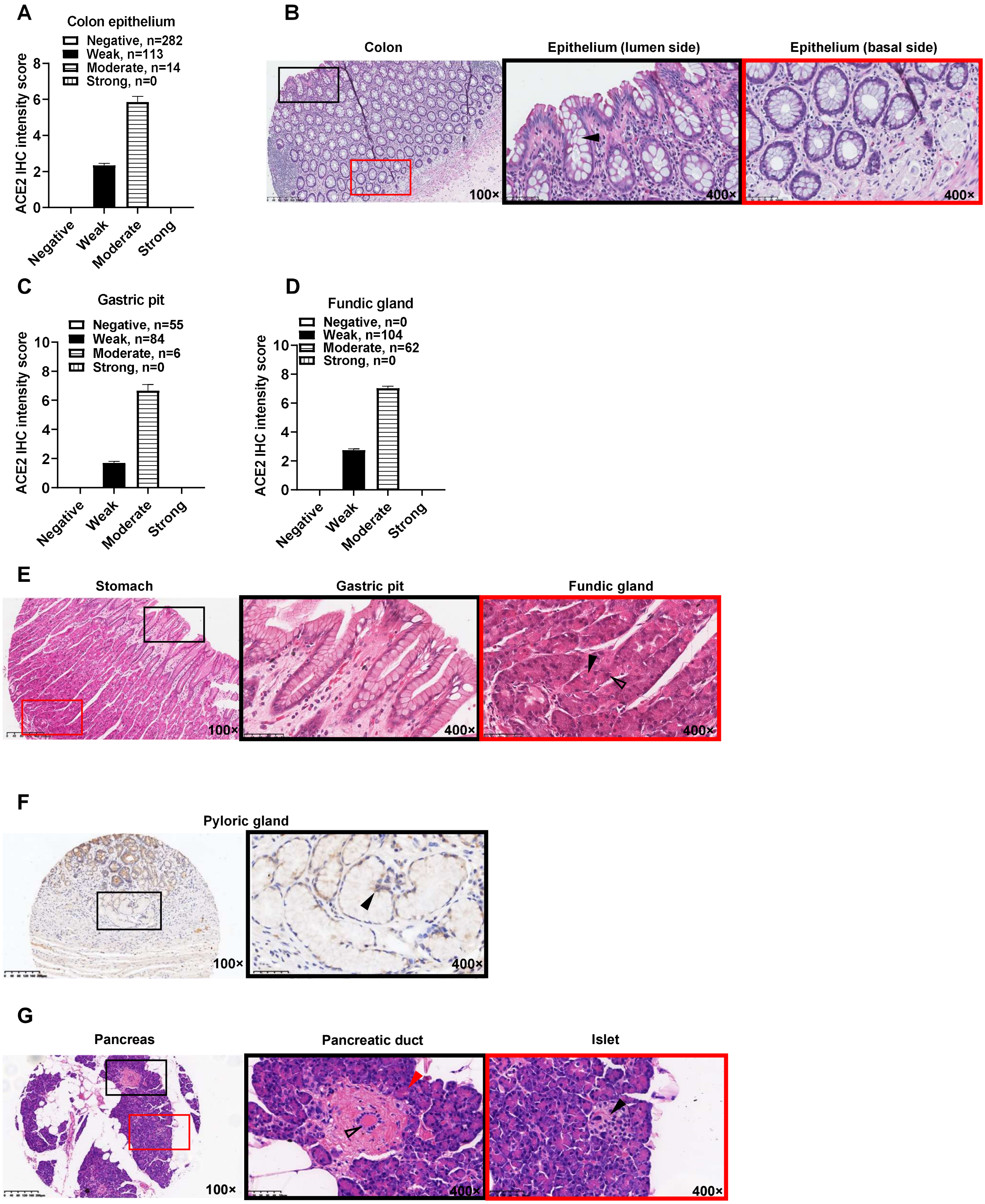

A
Colon epithelium
Negative, n=282
Weak, n=113
Moderate, n=14
Strong, n=0
ACE2 IHC intensity score
Negative
Weak
Moderate
Strong
B
Colon
Epithelium (lumen side)
Epithelium (basal side)
100×
400×
400×
C
Gastric pit
Negative, n=55
Weak, n=84
Moderate, n=6
Strong, n=0
ACE2 IHC intensity score
D
Fundic gland
Negative, n=0
Weak, n=104
Moderate, n=62
Strong, n=0
ACE2 IHC intensity score
E
Stomach
Gastric pit
Fundic gland
100×
400×
400×
F
Pyloric gland
100×
400×
G
Pancreas
Pancreatic duct
Islet
100×
400×
400×

Fig. S2

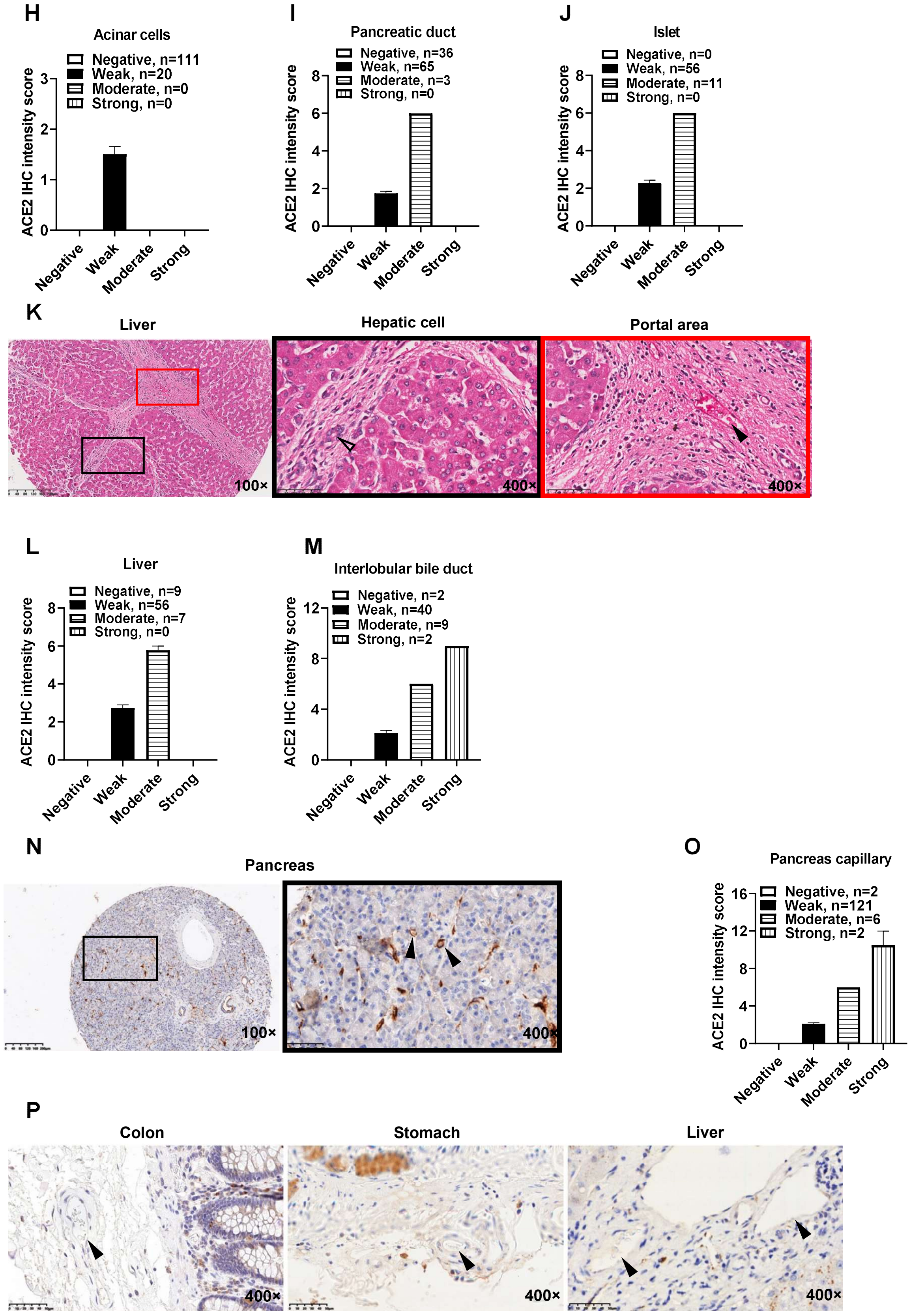

Figure S2. Expression profiles of ACE2 in gastrointestinal tissues. (A) Expression profile of ACE2 in colon epithelium**. (B)** Representative H&E images of colon epithelium (black arrow: goblet cell). **(C)** Expression profile of ACE2 in gastric pit**. (D)** Expression profile of ACE2 in stomach fundic gland**. (E)** Representative H&E images of stomach (hollow arrow: chief cells, black arrow: parietal cells). **(F)** Representative IHC

staining of pyloric gland with anti-ACE2 antibody. **(G)** Representative H&E images of pancreas**.** Acinar cells (red arrow), pancreas duct (hollow arrow) and pancreatic islet (black arrow). **(H-J)** Expression profile of ACE2 in acinar cells **(H)**, pancreatic duct **(I)**, and pancreatic islet **(J). (K)** Representative H&E images of liver: interlobular duct (hollow arrow), interlobular vessel (black arrow). **(L-M)** Expression profile of ACE2 in liver **(L)** and liver interlobular bile duct **(M)**. **(N)** Representative IHC staining of human pancreas sections with anti-ACE2 antibody. The black arrows were indicated capillary between acinar cells. **(O)** Expression profile of ACE2 in pancreas capillary**. (P)** Representative IHC staining of human colon, stomach, and liver sections with anti-ACE2 antibody. The black arrows were indicated capillary**.** Scale bar, 200μm (100×), 50μm (400×).Data are shown as mean ± s.e.m **(A, C, D, H, I, J, L, M, O).**

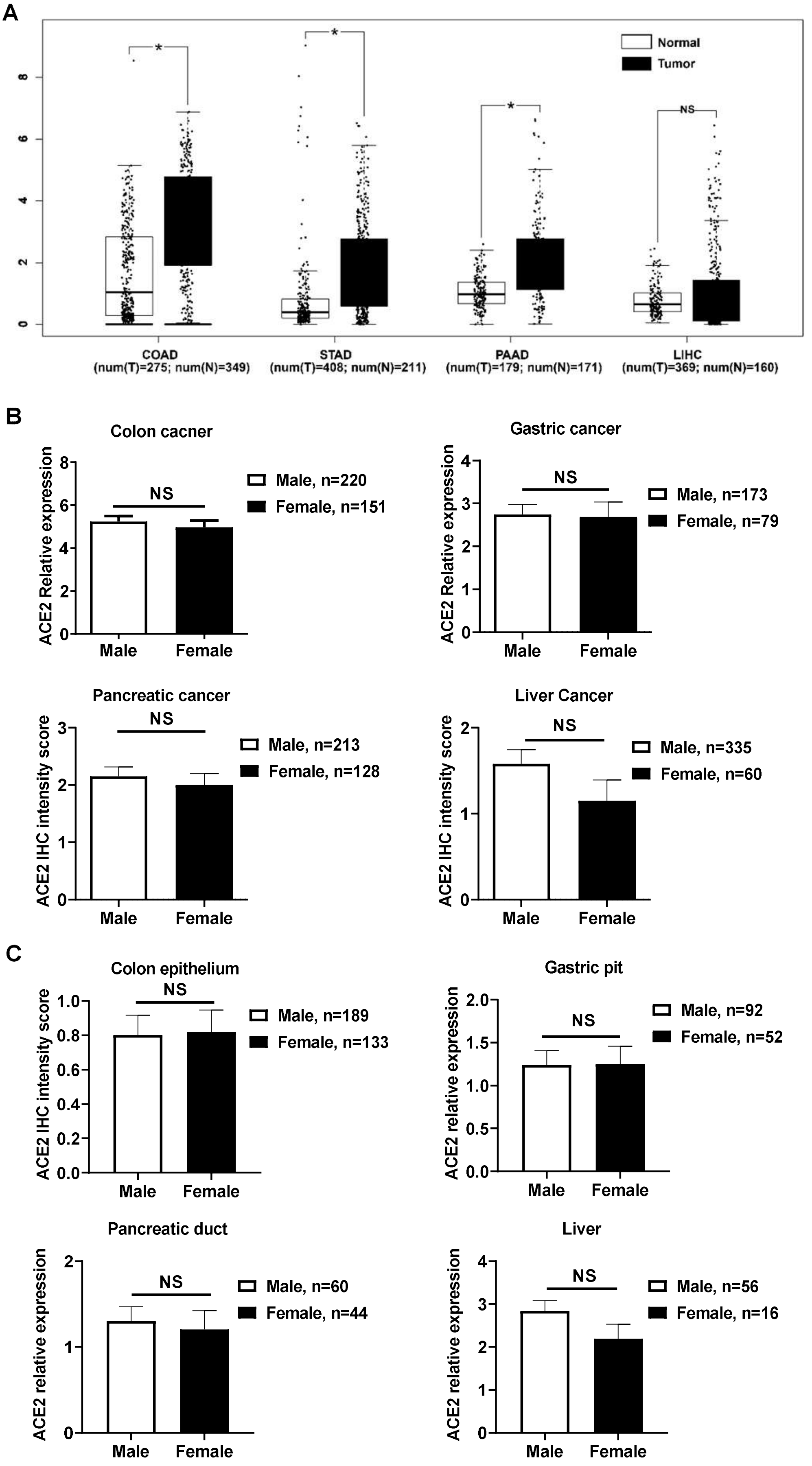

Figure S3. Expression profiles of ACE2 in gastrointestinal tumor tissues and normal tissues. (A) mRNA expression of ACE2 levels in colon adenocarcinoma (COAD), stomach adenocarcinoma (STAD), pancreatic adenocarcinoma (PAAD), and liver hepatocellular carcinoma (LIHC) from GEPIA (Gene Expression Profiling

Interactive Analysis) based on TCGA (The Cancer Genome Atlas) database and Genotype-Tissue Expression (GTEx) project. **(B-C)** Comparison of ACE2 protein expression in gastrointestinal tumor tissues **(B)** or adjacent non-tumor gastrointestinal tissues **(C)** between male and female. Data are shown as mean ± s.e.m **(A, B, C)**. NS, no significance.

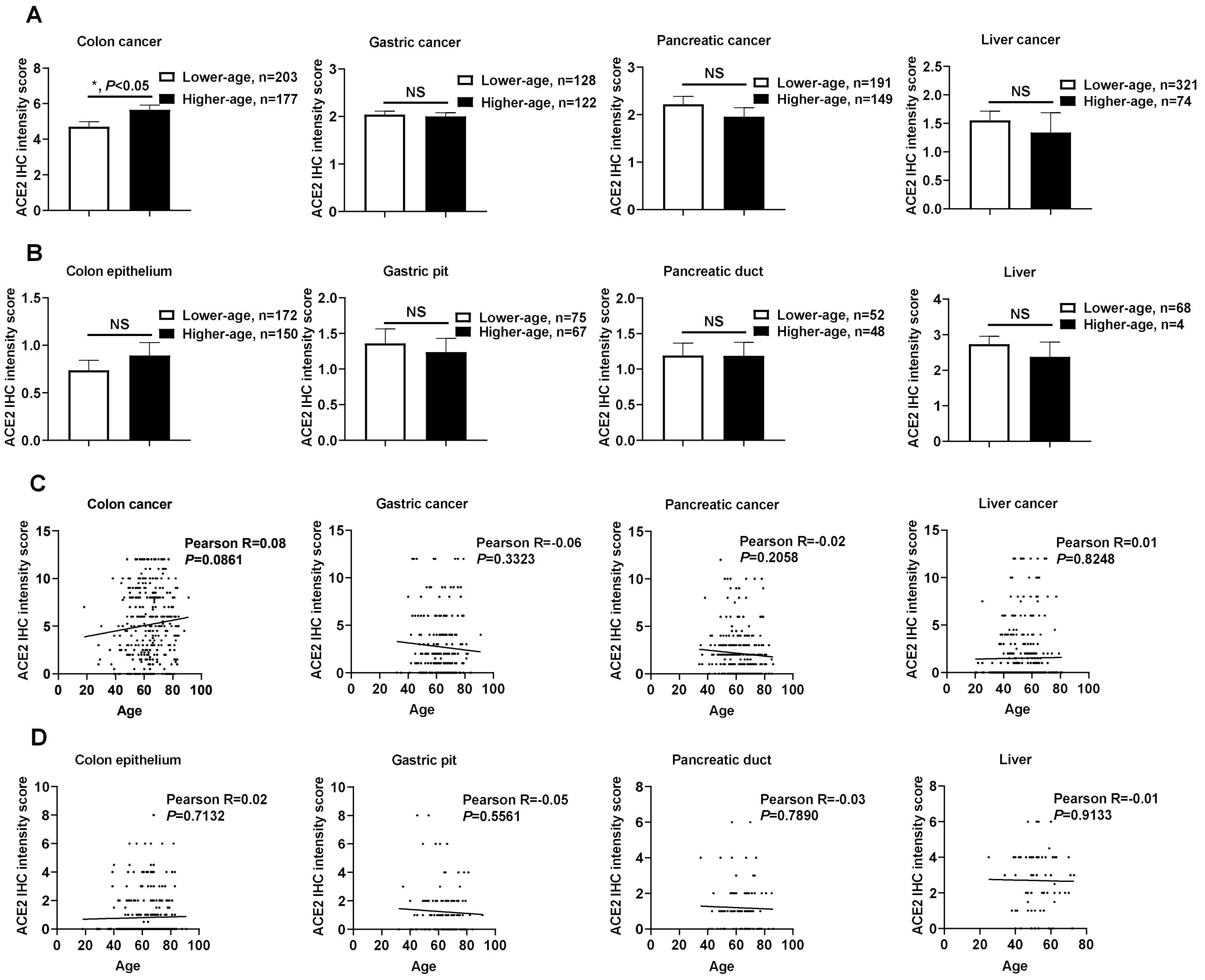

Figure S4. The age association in the ACE2 protein in gastrointestinal tissues. Patients were divided into two groups: Lower-age (< 65 years) and Higher-age (≥ 65 years). (A) ACE2 expression profiles in Lower-age or Higher-age patients with gastrointestinal cancers. **(B)** ACE2 expression profiles in adjacent non-tumor gastrointestinal tissues from Lower-age or Higher-age patients. **(C-D)** Correlation of ACE2 expression with age in gastrointestinal tumor tissues **(C)** or in adjacent non-tumor tissues **(D)**. Data are shown as mean ± s.e.m **(A, B)**. Each symbol represents one patient **(C, D)**. NS, no significance.

Fig. S5

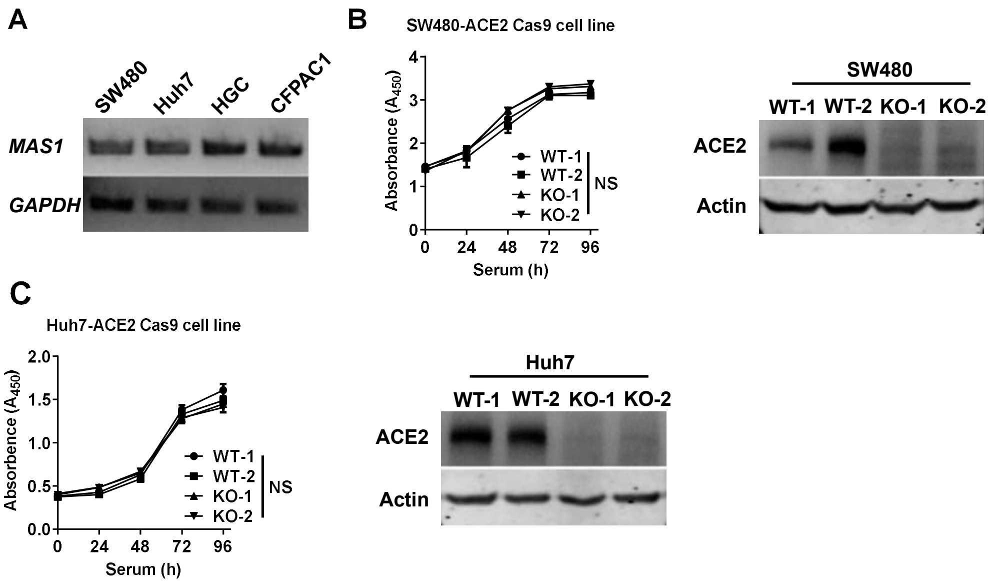

Figure S5. ACE2 does not affect the proliferation of gastrointestinal tumor cell lines. (A) *MAS1* mRNA expression in SW480, Huh7, HGC and CFPAC1 cells. **(B)** The proliferation of the ACE2 wide type (WT) and ACE2 knockout (KO) SW480 cells. Western blotting showed ACE2 knockout efficiency in SW480 cells. **(C)** The proliferation of the ACE2 wide type (WT) and ACE2 knockout (KO) Huh7 cells. Western blotting showed ACE2 knockout efficiency in Huh7 cells. Data are shown as mean ± s.e.m and are representative of three independent experiments. NS, no significance.

Table S1 Clinical characteristics of colon cancer patients

Variable	ACE2 IHC intensity				*p*-Value
	Negative n (%)	Weak n (%)	Moderate n (%)	Strong n (%)	
**Gender**					0.245
Male	34 (15.4)	60 (27.3)	71 (32.3)	55 (25.00)	
Female	23 (15.2)	49 (32.5)	45 (29.8)	34 (22.5)	
**Age (years)**					0.054
Median	61	62	66	61	
Range	39-81	28-84	18-91	29-82	
**T stage**					0.815
T1	1 (10.0)	2 (20.0)	4 (40.0)	3 (30.0)	
T2	11 (21.6)	11 (21.6)	17 (33.3)	12 (23.5)	
T3	39 (13.5)	84 (29.2)	92 (31.9)	73 (25.4)	
T4	5 (17.2)	13 (44.8)	8 (27.6)	3 (10.4)	
**N stage**					0.879
N0	33 (17.1)	45 (23.3)	64 (33.2)	51 (26.4)	
N1	14 (10.8)	48 (36.9)	41 (31.5)	27 (20.8)	
N2	9 (16.4)	17 (30.9)	16 (29.1)	13 (23.6)	
**M stage**					0.030
M0	52 (14.7)	96 (27.2)	115 (32.6)	90 (25.5)	
M1	4 (15.4)	14 (53.8)	7 (26.9)	1 (3.9)	
**TNM stage**					0.021
I	11 (17.5)	14 (22.2)	21 (33.3)	17 (27.0)	
II	23 (16.1)	38 (26.6)	45 (31.5)	37 (25.8)	
III	18 (11.0)	54 (33.1)	55 (33.8)	36 (22.1)	
IV	4 (44.4)	4 (44.4)	0 (0)	1 (11.2)	

p value < 0.05 was considered to indicate statistical significance. **T stage**, primary tumor; **N stage**, regional lymph nodes; **M stage**, distant metastasis.

Table S2 Clinical characteristics of gastric cancer patients.

Variable	ACE2 IHC intensity				*p*-Value
	Negative n (%)	Weak n (%)	Moderate n (%)	Strong n (%)	
**Gender**					0.927
Male	49 (28.3)	86 (49.7)	22 (12.7)	16 (9.3)	
Female	21 (26.6)	41 (51.9)	12 (15.2)	5 (6.3)	
**Age (years)**					0.595
Median	65	65	62	66	
Range	32-84	45-91	40-78	44-79	
**T stage**					0.004
T1	3 (21.4)	9 (64.3)	2 (14.3)	0 (0.00)	
T2	8 (47.1)	7 (41.2)	0 (0)	2 (11.7)	
T3	29 (47.5)	21 (34.4)	8 (13.2)	3 (4.9)	
T4	29 (18.2)	90 (56.6)	24 (15.1)	16 (10.1)	
**N stage**					0.150
N0	24 (38.6)	31 (50.0)	6 (9.9)	1 (1.5)	
N1	45 (20.5)	28 (63.6)	5 (11.4)	2 (4.5)	
N2	36 (29.3)	68 (43.1)	23 (15.5)	18 (12.1)	
N3	19 (21.8)	43 (49.4)	14 (16.1)	11 (12.4)	
**M stage**					0.761
M0	66 (28.1)	119 (50.6)	30 (12.8)	20 (8.5)	
M1	4 (23.53)	8 (47.06)	4 (23.53)	1 (5.88)	
**TNM stage**					0.109
I	6 (31.6)	11 (57.9)	2 (10.5)	0 (0)	
II	24 (40.7)	28 (47.5)	5 (8.5)	2 (3.3)	
III	36 (22.8)	80 (50.6)	24 (15.2)	18 (11.4)	
IV	4 (25.0)	8 (50.0)	3 (18.8)	1 (6.2)	
**Lauren classification**					0.503
Intestinal	24 (22.2)	66 (61.1)	14 (13.0)	4 (3.7)	
Diffuse	45 (32.4)	59 (42.5)	18 (12.9)	17 (12.2)	
Mix	1 (14.3)	4 (57.1)	2 (28.6)	0 (0)	

p value < 0.05 was considered to indicate statistical significance. **T stage**, primary tumor; **N stage**, regional lymph nodes; **M stage**, distant metastasis.

Table S3 Clinical characteristics of pancreatic cancer patients.

Variable	ACE2 IHC intensity				*p*-Value
	Negative n (%)	Weak n (%)	Moderate n (%)	Strong n (%)	
**Gender**					0.494
Male	61 (28.6)	128 (60.1)	16 (7.5)	8 (3.8)	
Female	38 (29.7)	77 (60.1)	9 (7.1)	4 (3.1)	
**Age (years)**					0.263
Median	65	62	61	60	
Range	41-85	34-86	38-79	49-79	
**T stage**					0.557
T1	8 (42.1)	8 (42.1)	1 (5.3)	2 (10.5)	
T2	40 (27.2)	94 (64.0)	9 (6.1)	4 (2.7)	
T3	33 (26.6)	81 (65.3)	7 (5.7)	3 (2.4)	
T4	1 (12.5)	6 (75.0)	0 (0)	1 (12.5)	
**N stage**					0.566
N0	36 (23.4)	102 (66.2)	11 (7.1)	5 (3.3)	
N1	42 (29.2)	91 (63.2)	7 (4.8)	4 (2.8)	
N2	4 (33.3)	5 (41.7)	1 (8.3)	2 (16.7)	
**M stage**					0.706
M0	81 (26.7)	195 (64.1)	18 (5.9)	10 (3.3)	
M1	4 (28.6)	8 (57.2)	1 (7.1)	1 (7.1)	
**TNM stage**					0.929
I	22 (27.8)	51 (64.6)	3 (3.8)	3 (3.8)	
II	55 (27.8)	124 (62.6)	14 (7.1)	5 (2.5)	
III	4 (25.0)	10 (62.5)	0 (0)	2 (12.5)	
IV	4 (28.7)	8 (57.1)	1 (7.1)	1 (7.1)	

p value < 0.05 was considered to indicate statistical significance. **T stage**, primary tumor; **N stage**, regional lymph nodes; **M stage**, distant metastasis.

Table S4 Clinical characteristics of liver cancer patients.

Variable	ACE2 IHC intensity				*p*-Value
	Negative n (%)	Weak n (%)	Moderate n (%)	Strong n (%)	
**Gender**					0.800
Male	218 (65.1)	72 (21.4)	28 (8.4)	17 (5.1)	
Female	36 (60.0)	20 (33.3)	4 (6.7)	0 (0)	
**Age (years)**					0.600
Median	56	57	57	58	
Range	21-81	22-79	40-77	45-69	
**T stage**					0.805
T1	6 (54.5)	2 (18.2)	2 (18.2)	1 (9.1)	
T2	12 (57.1)	8 (38.1)	0 (0)	1 (4.8)	
T3	23 (57.5)	14 (35.0)	1 (2.5)	2 (5.0)	
T4	0 (0)	0 (0)	0 (0)	0 (0)	
**N stage**					0.020
N0	40 (59.7)	21 (31.3)	3 (4.5)	3 (4.5)	
N1	1 (20.0)	3 (60.0)	0 (0)	1 (20.0)	
**M stage**					-
M0	41 (56.9)	24 (33.3)	3 (4.2)	4 (5.6)	
M1	0 (0)	0 (0)	0 (0)	0 (0)	
**TNM stage**					0.096
I	133 (68.2)	38 (19.5)	18 (9.2)	6 (3.1)	
II	55 (61.8)	20 (22.5)	9 (10.1)	5 (5.6)	
III	22 (70.9)	6 (19.4)	2 (6.5)	1 (3.2)	
IV	3 (42.9)	3 (42.9)	0 (0)	1 (14.2)	

p value < 0.05 was considered to indicate statistical significance. **T stage**, primary tumor; **N stage**, regional lymph nodes; **M stage**, distant metastasis.

Table S5 TNM staging (AJCC)

		Colon cancer	Gastric cancer	Liver cancer	Pancreatic cancer
Primary tumor (T)	T1	Tumor invades submucosa	Tumor invades submucosa	Solitary tumor ≤2cm with or without vascular invasion; solitary tumor >2cm without vascular invasion	Tumor limited to the pancreas, ≤ 2cm
	T2	Tumor invades muscularis propria	Tumor invades muscularis propria	Solitary tumor >2cm with vascular invasion or multifocal tumors none >5cm	Tumor >2 cm and ≤ 4cm
	T3	Tumor invades through muscularis propria into subserosa	Tumor invades through muscularis propria into subserosa	Multifocal tumors at least one of which is >5cm single	Tumor > 4cm
	T4	Tumor directly invades other organs or structure and/or perforate visceral peritoneum	Tumor directly invades other organs or structure and/or perforate visceral peritoneum	Tumor or multifocal tumors of any size involving a major branch of the portal vein of hepatic vein or tumor with direct invasion of adjacent organs other than the gallbladder or with perforation of visceral peritoneum	Tumor involves celiac axis, superior mesenteric artery, and/or common hepatic artery, regardless of size
Regional lymph nodes (N)	N0	No	No	No	No
	N1	1 to 3	1 to 2	Regional lymph node metastasis	1 to 3
	N2	4 or more	3 to 6	-	4 or more
	N3	-	7 or more	-	
Distant metastases (M)	M0	No	No	No	No
	M1	Distant metastasis	Distant metastasis	Distant metastasis	Distant metastasis
TNM stage	I	T1/T2, N0, M0	T1, N0/N1, M0; T2, N0, M0	T1, N0, M0	T1/T2, N0, M0
	II	T3/T4, N0, M0	T1, N2, M0; T2, N1, M0; T3, N0, M0; T1, N3, M0; T2, N2, M0; T3, N1, M0;	T2, N0, M0	T3, N0, M0; T1/T2/T3, N1, M0
	III	AnyT, N1/N2, M0	AnyT, N3, M0; T4, AnyN, M0; T3, N2, M0	T3/T4, N0. M0	AnyT, N2, M0; T4, AnyN, M0
	IV	AnyT, AnyN, M1	AnyT, AnyN, M1	AnyT, N1, M0; AnyT, AnyN, M1	AnyT, AnyN, M1
